# An intelligent neural network model to detect red blood cells for various blood structure classification in microscopic medical images

**DOI:** 10.1016/j.heliyon.2024.e26149

**Published:** 2024-02-13

**Authors:** Riaz Ullah Khan, Sultan Almakdi, Mohammed Alshehri, Amin Ul Haq, Aman Ullah, Rajesh Kumar

**Affiliations:** a Yangtze Delta Region Institute (Huzhou), University of Electronic Science and Technology of China, Huzhou 313001, China; b Department of Computer Science, College of Computer Science and Information systems, Najran University, Najran 55461, Saudi Arabia; c School of Computer Science and Engineering, University of Electronic Science and Technology of China, Chengdu 611731, China

**Keywords:** RBC detection, Object detection, Image processing, Deep learning

## Abstract

Biomedical image analysis plays a crucial role in enabling high-performing imaging and various clinical applications. For the proper diagnosis of blood diseases related to red blood cells, red blood cells must be accurately identified and categorized. Manual analysis is time-consuming and prone to mistakes. Analyzing multi-label samples, which contain clusters of cells, is challenging due to difficulties in separating individual cells, such as touching or overlapping cells. High-performance biomedical imaging and several medical applications are made possible by advanced biosensors. We develop an intelligent neural network model that can automatically identify and categorize red blood cells from microscopic medical images using region-based convolutional neural networks (RCNN) and cutting-edge biosensors. Our model successfully navigates obstacles like touching or overlapping cells and accurately recognizes various blood structures. Additionally, we utilized data augmentation as a pre-processing method on microscopic images to enhance the model's computational efficiency and expand the sample size. To refine the data and eliminate noise from the dataset, we utilized the Radial Gradient Index filtering algorithm for imaging data equalization. We exhibit improved detection accuracy and a reduced model loss rate when using medical imagery datasets to apply our proposed model in comparison to existing ResNet and GoogleNet models. Our model precisely detected red blood cells in a collection of medical images with 99% training accuracy and 91.21% testing accuracy. Our proposed model outperformed earlier models like ResNet-50 and GoogleNet by 10-15%. Our results demonstrated that Artificial intelligence (AI)-assisted automated red blood cell detection has the potential to revolutionize and speed up blood cell analysis, minimizing human error and enabling early illness diagnosis.

## 1 Introduction

The analysis of microscopic medical images has grown significantly in significance recently for the diagnosis and management of a variety of medical conditions. The identification and categorization of blood cells are key components of these disorders, particularly when it comes to diseases of the blood. However, manually identifying cells in these minute images is a lot of work and can occasionally result in errors. Researchers have concentrated on creating powerful neural network models that can automatically detect and classify red blood cells based on various blood structure classifications, utilizing the capabilities of new biosensors. This is done in an effort to overcome these issues. Using deep learning techniques, our models reliably identify and categorize red blood cells based on their structural characteristics. By incorporating state-of-the-art biosensors and deep learning algorithms into our models, we hope to increase the accuracy and efficiency of blood cell analysis in microscopic medical images. This research could significantly influence medical diagnostics and advance customized healthcare. The creation of such models has the potential to transform the way blood cells are analyzed in microscopic medical imaging, saving time and lowering the chances of errors that medical professionals would make. This innovative approach ultimately leads to more accurate diagnoses and improved patient outcomes. For instance, the evaluation of a complete blood count test and the examination of blood samples are crucial diagnostic tools in identifying anemia, malaria, and leukemia, illuminating the significance of accurate red blood cell detection and classification in research and the diagnosis of blood-related disorders. The Complete Blood Count (CBC) is a common procedure for determining the concentration and composition of all blood components, including platelets, Red Blood Cells (RBC), White Blood Cells (WBC), hemoglobin levels, and mean red blood cell volumes. This test is one of many that make up a thorough physical examination. [1].

In working towards automatic detection of red blood cells, assessments of blood smears play a key role in diagnosis. Microscopic images can be utilized for the early detection, investigation, and measurement of several blood illnesses when specific parasites like Babesia and Malaria directly infect red blood cells (RBCs) [2]. Nonetheless, manually or visually assessing WBCs in leukemia and parasitemia in thinner blood films is a tiresome process that is susceptible to errors in counting [3]. Developing algorithms capable of precisely segmenting and enumerating red blood cells in microscopy images, and furnishing data on the distribution of minute particles, would be advantageous in ensuring precise clinical analysis. For instance, Khalid [4] and Rusul [5] presented transfer learning-based models for automatically diagnosing skin cancer and brain strokes because manual diagnosis is a challenging task for human beings due to identical properties in images. Similarly, deep learning models are important to automatically diagnose abnormalities in blood cells. If there is a red blood cell abnormality for example anemia, more indices of red blood cells are examined, including Mean Cell Volume (MCV), Mean Cell Hemoglobin (MCH), Red Blood Cell Distribution Width (RDW), Red Blood Cell (RBC) and Mean Corpuscular Hemoglobin Concentration (MCHC), to help identify the underlying causes. Normally, there are 4.2 million to 5.9 million RBCs per square centimeter in healthy blood. The typical range for White Blood Cell (WBC) counts in normal blood is 4,500 to 10,000 WBCs per microliter [6]. An elevated white blood cell (WBC) count, exceeding 30,000 cells per microliter, can indicate the presence of various conditions such as systemic illness, infection, inflammation, leukemia, allergy, or tissue damage from burns [7]. In addition to red and white blood cell abnormalities, platelet counts also provide important diagnostic information. In cases of lower platelets, for example with dengue infection, platelet levels are closely observed. If platelet counts fall below critical levels, patients may require platelet transfusions [7].

The main objective of this research is to propose a comprehensive framework for the accurate identification of red blood cells in imaging data. Through the use of CBC tests on blood smear images, this framework has the potential to significantly improve the evaluation, diagnosis, and monitoring of a wide range of medical problems, such as anemia, leukemia, infections, and allergic reactions. Additional red blood cell indicators are analyzed in cases of anomalies, such as anemia, to determine the underlying causes. The research presented here introduces a multi-model method for the detection of red blood cells that is based on deep learning technology. The deep learning component consists of pre-processing, feature extraction, and image classification tasks [8]. Region-based Convolutional Neural Network (RCNN) was used to accurately identify the area of interest by proficiently characterizing RBCs in microscopic images. The subsequent are the key contributions of this research.

1. We presented a framework by incorporating advanced biosensors and deep learning algorithms into our models, we aim to enhance the accuracy and efficiency of blood cell analysis in microscopic medical images.
2. The Radial Gradient Index filtering algorithm was modified regarding the removal of noise data for microscopic images based on data normalization.
3. Data augmentation technique is utilized to improve the resolution and solve the data imbalanced problem.
4. For the characterization and effective detection of RBCs, an RCNN model has been executed.

Section 2 of this paper provides a summary of prior research on the counting and segmentation of RBCs. Section 3 presents the methodology and proposed framework based on neural network. Section 4 analyzes the accuracy, and time and identifies the RBC samples, also brief discussion on the experiments and the results. Section 5 discusses the limitations of the current research and gives future directions in the relevant field. Lastly, Section 6 is concluding the paper.

## 2 Related work

Recently, the field of artificial neural networks has been progressing rapidly, especially with the invention of innovative machine and deep learning algorithms [9]. Deep learning has emerged from artificial neural networks. The learning procedure of deep learning is inspired by the human brain's learning process. It can be used to learn the approximate unknown function from the deep nonlinear network structure. In addition, deep learning learns from the characteristics of the sample dataset. Deep learning has found many vertical applications in various fields such as image, handwritten numerals, and speech recognition.

There have been several recent research studies focused on the categorization and enumeration of blood cells. Gangadhar et al., [10], described the development of an automated system for categorizing and enumerating blood cells using holographic imaging and deep learning algorithms. The automated system demonstrated an impressive overall accuracy of 97.4% in classifying various blood cell types, which include red blood cells, white blood cells, and platelets. Specifically, for red blood cell quantification, the system achieved a remarkable 98.2% accuracy, coupled with a precision of 98.5% and a recall of 97.8%. In the case of white blood cell enumeration, the system attained an accuracy rate of 96.1%, precision at 96.7%, and recall of 95.2%. For platelet counting, the system excelled with an accuracy of 98.9%, a precision of 99.1%, and a recall of 98.6%. Moreover, the automated counts exhibited a strong correlation with counts conducted manually by experts, boasting R2 values of 0.98, 0.96, and 0.99 for red blood cells, white blood cells, and platelets, respectively. Remarkably, the system maintained consistent performance across multiple blood samples, with accuracy variability remaining below 5

Another study published in 2022 by Sharma et al., [11], utilized deep learning algorithms for the classification and enumeration of blood cells. The study demonstrated that highly accurate and specific deep-learning algorithms could count and classify several kinds of blood cells. A Deep Learning model, utilizing DenseNet121, was employed for the classification of different white blood cell types in blood cell images. This model exhibited outstanding performance, achieving an accuracy of 98.84%, precision of 99.33%, sensitivity of 98.85%, and specificity of 99.61%.

Raillon Camille et al., [12], concentrated on the cataloging and classification of Circulating Tumor Cells (CTCs) in the blood. The study showed that this approach could precisely distinguish and categorize CTCs from other blood cells by isolating and analyzing individual CTCs using microfluidic single-cell analysis. Gaps in several of the identified RBCs suggested that more preprocessing steps were needed to improve the accuracy of the findings. These findings illustrate the precision of the impedance chip in enumerating both beads and cancer cells, resulting in a mean counting error of 1.0% for beads and 3.5% for cancer cells. Furthermore, the size-based categorization of beads of varying dimensions was effectively accomplished, highlighting the impedance chip's capacity to distinguish cancer cells from smaller blood cells based on size-related data.

Jung et al., [13], created a model to count RBCs and detect malarial parasites automatically in thinner blood samples. They employed Otsu thresholding to separate the infected RBCs after processing a layer of green color to count the RBCs. In the above studies, holographic imaging was not discussed, therefore the dataset may contain errors or other problems that make it difficult to identify cells. For example, the methods these studies employed were not capable of identifying cells that were clustered together or overlapping. The system's exceptional limit of detection, capable of identifying parasites with a mere probability of 0.00066112%, highlights its remarkable sensitivity. Additionally, the system attained perfect 100% sensitivity and specificity, accurately recognizing all P. vivax and P. falciparum samples. These results emphasize the system's potential for swift malaria diagnosis and monitoring of parasite density, providing substantial benefits over conventional microscopy.

Cruz Dela et al. [14] presented a methodology to count RBCs, WBCs, and platelets. They performed several preprocessing processes before the image is converted to binary and proceeded on to cell segmentation and enumeration based on an ideal threshold value obtained from a histogram. Despite achieving a 95% accuracy rate, their approach was not able to identify cells that were overlapping. Additionally, the use of iterative thresholds may lead to the elimination of pertinent data, leading to a reduction in segmentation accuracy.

In order to tackle the issue of overlapping cells, Nenden et al., [15], employed distance transform and proposed a technique that specifically targeted clustered cells. Initially, they determined central points by means of distance transformation. The most favorable central points were chosen based on the extent of borderline coverage and the average cell size was estimated via single-cell extraction. They then developed an algorithm that employed a single-cell mask to separate cells. Experts annotated the ground truth for their dataset, and the proposed method demonstrated an accuracy of 96% and 70% when employing the Distance Transform method. However, the limitation of their study was that their method only tolerated regularly shaped and high magnification-focused cells, whereas not all blood cells possess such regular shapes, particularly if they are diseased. Hence, it is important for cell detection methods to be capable of detecting irregularly shaped cells. Moreover, their method's performance was impacted by noise, resulting in sub-optimal results.

Chen and Chung [16] proposed a circular detection technique known as Randomized Circle Detection (RCD), claiming that it outperforms other Hough Transform-based algorithms in terms of efficiency. Unlike other algorithms that use accumulators, RCD selects four edge pixels randomly from the images and examines them for non-collinearity to form a candidate circle. The algorithm then resolves whether the circle is a possible one on the basis of the distance criterion. The algorithm then counts the pixels that are within the circle's boundaries after determining its center and radius. This is accomplished by measuring the gap between every pixel in the image and the border of the potential circle, and if the gap falls below a predetermined level, the pixel is deemed to be part of the circle's boundary. However, RCD has certain drawbacks, including low efficiency while working with huge images with a lot of edge pixels and a decreased chance of generating a genuine circle because only four edge pixels are randomly chosen. RCD is less effective in detecting irregular circles because it requires a lot of predefined parameters and threshold values, and has a fixed number of iterations that are correlated significantly with pixel intensity.

Due to the Hough Transform's effective performance, researchers have utilized it for circle detection in microscopic images for RBC and WBC calculation tasks [17], [18]. Mahmood et al. [18] employed the Hough Transform to count WBCs and RBCs in microscopic images. The first step involved converting the original image to a color image structure and color-segmenting the white and red cells depending on the feature luminance over through the channels spanning from 130 to 150 and 80 to 100, respectively. They used some segmentation algorithms in the second stage, then the Canny operator for the edge detection procedure. The final step involved employing the Hough Transform for detection and counting cells. The accuracy of the results varied from 64% to 87%, reliant on the cell types being analyzed and the processing methods employed. However, the Hough Transform method necessitated significant memory storage and computational time to determine an extensive range of accepted radii. Moreover, it had a limited tolerance for uneven and highly overlapping cells, which reduced its accuracy.

Mahmood and Mansor [19] analyzed ten sample images of normal cells by first transforming the image to the HSV color space and selecting the Saturation channel, or “S”, for further analysis. The cells were divided using wavelet transforms and adaptive threshold techniques over the S channel. The method proposed by them achieved an accuracy rate of approximately 96% in comparison to manual counting. However, this approach is suitable for simple cases where detected cells are normal, and the amount of cells that overlap is small with a regular shape. In such cases, the Hough Transform can significantly contribute to achieving good performance.

Venkatalakshmi and Thilagavathi [20] employed a comparable technique to Mahmood and Mansor [19] to count RBCs in microscopic images utilizing Circular Hough Transform. However, their method, like Mahmood and Mansor's approach, was less flexible when dealing with overlapping or oddly shaped cells. In [21], a filter was introduced to eliminate segmentation errors and smooth the edges of images. Shan et al. [22] presented a lesion detection method that exploits speckle reduction and considers both spatial and texture features, using anisotropic diffusion for speckle reduction. Diaz-Pernil et al. [23] emphasized bio-inspired parallel applications for exploring homology geometrical objects in 2D images. However, these methods are often limited in terms of feature extraction and do not sufficiently analyze important features.

In contrast to various related studies discussed in this paper, which commonly assess performance using metrics such as detection accuracy, precision, and recall, this paper adopts a distinct approach. It leverages a specific deep learning model, RCNN, designed for region-based detection, setting it apart from other works that rely on different model architectures like CNNs and autoencoders. Notably, the innovative aspect lies in the integration of biosensors into the RCNN model, distinguishing it from papers that predominantly employ off-the-shelf deep networks like ResNet and GoogLeNet. In terms of preprocessing, this paper employs a tailored filtering algorithm, a departure from the standard techniques like thresholding and morphological operations seen in some prior works. Additionally, this paper introduces data augmentation to expand the dataset, a feature often absent in most related works. Another significant advantage of RCNN is its enhanced capability to handle overlapping and irregularly shaped cells, a task that Hough Transform in previous works may not address as effectively. By utilizing a customized RCNN model with biosensor integration and unique preprocessing techniques, this methodology is positioned as a more advanced and differentiated approach compared to related works that predominantly employ more generic techniques. The focus on handling overlapping cells also differentiates this approach.

## 3 Proposed model

**Figure 1:**
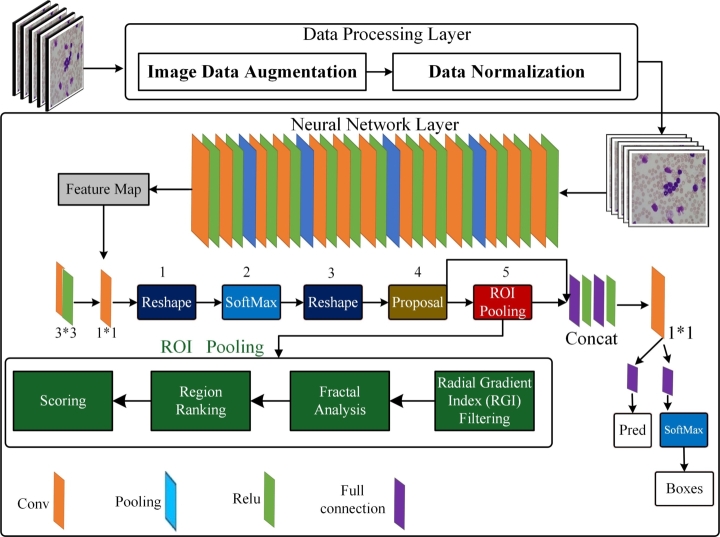
Graphical Representation of the Proposed Model for Red Blood Cell Detection.

The following section outlines the RBC techniques that involve two distinct phases, namely, i) Data Processing and ii) RBC detection based on RCNN. To start with, the proposed data preprocessing methods involve the filtering algorithm, which enhances the image resolution while minimizing noise, and the data augmentation technique that boosts the model's computational efficiency and enhances the size of the sample. Furthermore, an RCNN is trained for detecting and characterizing RBCs. Our proposed framework retains important information and helps improve the accuracy of RBC detection. Fig. 1 describes the detailed architecture of the layers of the proposed model.

1. **Data Processing Layer**:
  (a) **Image Data Augmentation**: In our proposed model, the “Data Processing Layer” plays a crucial role. We utilize “Image Data Augmentation,” a common technique in deep learning, to artificially modify input images, such as rotation, scaling, and flipping. This augmentation enhances the diversity of our training data, helping our model generalize better.
  (b) **Data Normalization**: Additionally, data normalization is a key step. We scale pixel values to a standard range, typically between 0 and 1, to ensure faster convergence during training.
2. **Neural Network Layer**:
  (a) Our “Neural Network Layer” contains a series of operations, including Convolution (Conv), Pooling, Rectified Linear Units (Relu), and more. These operations progressively transform the input image data, extracting hierarchical features crucial for our model's performance. Each colored block represents an individual layer or operation in our neural network.
  (b) The specific operations like Reshape, SoftMax, Proposal, ROI Pooling, and Concat indicate different stages in our network's architecture, tailored to the requirements of our object detection task.
  (c) ROI Pooling (Region Of Interest Pooling) is an integral part of our model, particularly useful for resizing variable-sized regions of interest to a consistent size, making it an essential step in our object detection process.
3. **Reshape, SoftMax, Proposal, ROI (Region of Interest) Pooling**:
  (a) In the context of our neural network, “reshaping” is applied to adjust the dimensions of tensors without altering their data. This step is pivotal to align tensors with the input or output requirements of specific layers in our network, optimizing the data flow.
  (b) Softmax serves as the activation function in our model. It converts real-number vectors into probability distributions, a critical component in the output layer for classification tasks.
  (c) In our object detection framework, we employ the “Proposal” step to generate potential bounding boxes that may contain objects of interest. These proposals are based on specific criteria and are further processed by our neural network for object classification and precise bounding box coordinates.
  (d) In our model, we define “Region of Interest” (ROI) as proposed bounding boxes that potentially enclose objects we aim to detect. ROI Pooling is a fundamental element of our object detection process. It ensures that variable-sized ROIs are resized to a consistent format, facilitating subsequent analysis and classification.
4. **Post Neural Network Operations**:
  (a) Our “Post Neural Network Operations” encompass a range of processing steps aimed at refining the detections made by our neural network. These include “Scoring,” “Region Ranking,” “Fractal Analysis,” and “Radial Gradient Index (RGI) Filtering.” These post-processing steps enhance the accuracy and reliability of our object detection.
  (b) After these operations, we derive final predictions (Pred) and bounding boxes (Boxes), which provide valuable insights into the detected objects and their respective confidences.

### 3.1 Pre-processing the data and detecting RBCs in clinical images

In general, even the advanced techniques have limitations, particularly when it comes to image processing which heavily relies on rule-based techniques and assumptions. However, deep learning models have been shown to achieve high accuracy in object detection. Algorithm 1 describes the procedure of cleaning or de-noising the clinical microscopic images. This algorithm assumes a binary segmentation of the cells, where the cells are labeled as either 0 (background) or 1 (foreground). The morphological operations at the end of the algorithm are used to further refine the segmentation and remove any remaining noise.

**Algorithm 1:**
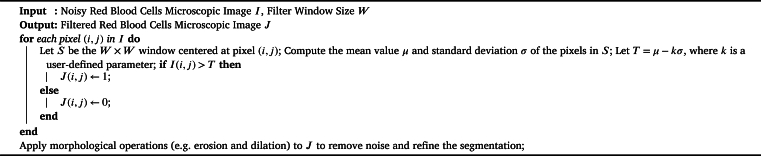
Enhance Images; Noise Reduction in Microscopic Images with Radial Gradient Index filtering algorithm.

Let *I* be a grayscale microscopic image of size m×n. We represent each pixel in the image by its intensity value I(i,j), where i=1,2,…,m and j=1,2,…,n.

Bilateral filtering works by smoothing the image while preserving edges. It achieves this by weighting the contributions of neighboring pixels based on both their distance and their similarity in intensity. The bilateral filter uses a Gaussian function to compute the weights for each pixel in the image.

Let w(i,j,p,q) be the weight assigned to pixel (p,q) when filtering pixel (i,j), where (i,j) and (p,q) are pixel coordinates. The weight *w* can be computed as:

(1) $$w(i,j,p,q)=w_{d}(i,j,p,q)\cdot w_{r}(i,j,p,q)$$

where $w_{d}$ is the distance weight and $w_{r}$ is the range weight.

The distance weight $w_{d}$ measures the spatial distance between pixels (i,j) and (p,q), and can be computed using a Gaussian function:

(2) $$w_{d}(i,j,p,q)=e^{-\frac{{\left(i-p\right)}^{2}+{\left(j-q\right)}^{2}}{2\sigma_{d}^{2}}}$$

where $\sigma_{d}$ is a parameter that controls the spatial extent of the filter.

The range weight $w_{r}$ measures the intensity similarity between pixels (i,j) and (p,q), and can be computed using another Gaussian function:

(3) $$w_{r}(i,j,p,q)=e^{-\frac{{\left(I(i,j)-I(p,q)\right)}^{2}}{2\sigma_{r}^{2}}}$$

where $\sigma_{r}$ is a parameter that controls the intensity range of the filter.

The filtered image $I^{\prime }$ can be obtained by averaging the intensity values of the neighboring pixels, weighted by their respective weights *w*:

(4) $$I^{\prime }(i,j)=\frac{1}{W(i,j)}\sum_{p=1}^{m}\sum_{q=1}^{n}w(i,j,p,q)I(p,q)$$

while W(i,j) is the sum of the weights for all neighboring pixels.

In Equation (1), the weight w(i,j,p,q) is computed as the product of the distance weight $w_{d}(i,j,p,q)$ and the range weight $w_{r}(i,j,p,q)$. Equation (2) defines the distance weight $w_{d}$, Equation (3) defines the range weight $w_{r}$, and Equation (4) describes the computation of the filtered image $I^{\prime }$.

The Radial Gradient Index filtering Algorithm 1 was used for the efficient use of separable kernels and recursive filtering techniques. The filter is applied iteratively to the image until the desired level of smoothing is achieved. The parameters $\sigma_{d}$ and $\sigma_{r}$ can be adjusted to control the amount of smoothing and edge preservation in the filtered image. The Radial Gradient Index filtering algorithm is a computer method used to improve or extract particular aspects from digital images. It works by computing the gradient at each pixel, which represents the rate of pixel value change, and then indexing this gradient information in a radial pattern around an image's focal point. The approach highlights or extracts specific visual elements, such as edges or textures, by using various filtering operations on the indexed gradient data, such as thresholding or convolution. This approach is useful for pre-processing images and enhancing the visibility of particular image details based on radial gradient analysis. It finds applications in image processing, computer vision, and pattern identification.

Algorithm 2 is used to label red blood cells in images. The algorithm describes that the RCNN model can detect red blood cells in the microscopic image and output their bounding box coordinates as detections. The filtering model is then used to remove false detections, and the remaining filtered detections are used to label the red blood cells in the output image. The labeling is done by setting the pixel values within the bounding box of each filtered detection to 1 in the output labeled image.

**Algorithm 2:**
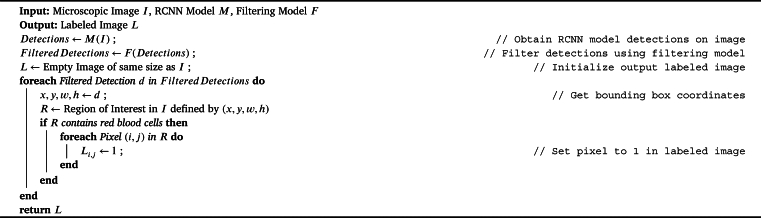
Labeling Red Blood Cells using RCNN Model with Filtering.

Let *I* be a grayscale microscopic image of size m×n. We represent each pixel in the image by its intensity value I(i,j), where i=1,2,…,m and j=1,2,…,n.

Image segmentation techniques are used to identify red blood cells in microscopic images. The division of an image into discernible sections, such as objects or backgrounds, is known as image segmentation. Using a threshold-based segmentation approach is one popular technique for red blood cell detection. In this method, the foreground (red blood cells) and background are initially distinguished by a threshold value of *T*. Then, we label each pixel as either foreground or background based on its intensity value as demonstrated in Equation (5):

(5) $$B(i,j)=\left\{ \begin{array}{cc} 1, & \text{if }I(i,j)>T\\ 0, & \text{otherwise} \end{array}\right.\;F(i,j)=\left\{ \begin{array}{cc} 1, & \text{if }I(i,j)\leq T\\ 0, & \text{otherwise} \end{array}\right.$$

where B(i,j) and F(i,j) demonstrate the binary images of the background and foreground, respectively.

After thresholding, we can further refine the segmentation by applying morphological operations, such as erosion and dilation. These operations can help to remove noise and small objects from the foreground and to connect nearby regions that belong to the same object.

Let *S* be a structuring element used for the morphological operations. The erosion and dilation operations can be defined as Equation (6) below:

(6) $$B_{\text{eroded}}(i,j)=\min_{(p,q)\in S}B(i+p,j+q)F_{\text{dilated}}(i,j)=\max_{(p,q)\in S}F(i+p,j+q)$$

where $B_{\text{eroded}}$ and $F_{\text{dilated}}$ are the binary images of the background and foreground, respectively, after applying erosion and dilation.

Finally, we can obtain the segmentation result by combining the binary images of the foreground and background:

(7) $$R(i,j)=\left\{ \begin{array}{cc} 1, & \text{if }F_{\text{dilated}}(i,j)=1\text{ and }B_{\text{eroded}}(i,j)=0\\ 0, & \text{otherwise} \end{array}\right.$$

where *R* represents the binary image of the red blood cells.

In Equation (5), the binary images of the background and foreground are defined after thresholding. In Equation (6), erosion and dilation operations are defined, and in Equation (7), the segmentation result is obtained by combining the binary images of the foreground and background.

**Figure 2:**
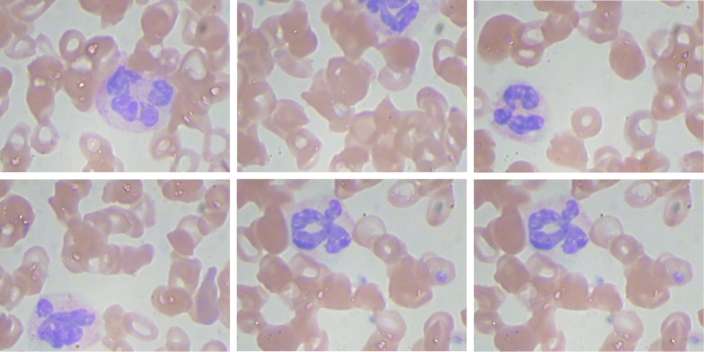
Samples of Clinical Microscopic Images.

In practice, the threshold value *T* and the structuring element *S* can be chosen based on the characteristics of the image and the desired level of segmentation accuracy. RCNN has rapidly developed as an interesting issue in image recognition and speech analysis. Its weight-sharing mechanism can significantly reduce parameter number and complexity. RCNN is particularly advantageous when dealing with multidimensional data, with images being the most common input parameter. RCNNs, unlike traditional techniques for feature extraction, are not impeded by the challenges of intricate feature extraction and data reconstruction. Multi-layer perceptron is designed to recognize features in 2-dimensional image data and can detect various transformations, such as scaling, translation, and rotation. To improve the identification performance, we are exploring an RCNN-based approach. In general, RCNNs consist of multiple stages for feature extraction, as shown in Fig. 1. The initial layer processes the input images and produces an image input layer that matches the dimensions of the training images. Afterward, convolution and subsampling layers are employed to extract and amplify features while minimizing data processing. Following this, the fully connected layer chooses relevant features and transforms 2D features into 1D, which gauges several classifier metrics. Ultimately, the classifier distinguishes cells based on their properties and categorizes them accordingly. The blood samples are shown in Fig. 2.

### 3.2 Training process of RCNN

The process of target detection in the general RCNN model consists of four stages. Firstly, suggested regions of interest inside the image are produced using the region-proposed technique. Subsequently, RCNN features are extracted from the regions, resulting in fixed-dimension features being output. Thirdly, the target is classified based on its characteristics. The RCNN eventually properly recognizes and merges ambient items using regression techniques in order to generate a precise object bounding box. In our proposed model scheme for precise object quantification and classification, we focus on the Region of Interest (ROI) in the image and combine various object features to detect RBC successfully. RCNN (Region-based Convolutional Neural Network) is a deep learning model used for object detection in images. The training process for RCNN is divided into the following steps:

1. **Dataset Preparation:** Initial step is to prepare the dataset for training. This includes collecting a large number of images that contain the objects or regions of interest, annotating the images with bounding boxes around the objects or regions, and dividing the dataset into training and testing sets. All input object anchors are assigned the measured feature map conv5-3, and the RPN network performs a forward estimation to extract anchor ratings and bounding box regression parameters.
2. **Feature Extraction:** RCNN uses a pre-trained CNN (Convolutional Neural Network) for feature extraction. The CNN is usually trained on a large dataset, and its weights are fixed during the training. The positions of proposal boxes are determined by using the anchored positions and the bounding box regression coefficients.
3. **Region Proposal:** Creating region recommendations for the objects in the image is the next stage. A selective search technique is used for this, producing a list of locations that are likely to contain objects. These regions are called region proposals. By setting the minimum and maximum values of the coordinates to 0 and the width or height of the image, respectively, the proposal coordinates that go beyond the image's borders are modified.
4. **Region of Interest (RoI) Pooling:** The region proposals are then passed through the CNN to extract features for each region. However, since each region proposal can be of a different size, it needs to be resized to a fixed size before passing it through the CNN. This is done using a technique called RoI pooling. Proposals that do not meet a specified size threshold (based on width and height) are filtered out.
5. **Classification:** The extracted features from each region proposal are then fed into a set of fully connected layers that classify the region proposal into one of the object categories or as background. After filtering, the remaining proposals are sorted in descending order based on their target score. Then, pre_nms_topN proposals (e.g. 6000) are extracted.
6. **Localization:** In addition to classifying the region proposal, RCNN also performs object localization by predicting the bounding box coordinates that tightly enclose the object within the region proposal. The extracted proposals undergo non-maximum suppression and the resulting proposals are filtered again to produce the final output. This is achieved by selecting the top post_nms_topN proposals (e.g. 300) based on their foreground score after nms.
7. **Training:** A multi-task loss function that combines the classification loss with the localization loss is used to train the RCNN. Whereas the localization loss evaluates the discrepancy between the expected and actual bounding box coordinates, the classification loss evaluates the variance between the anticipated and actual item classifications.
8. **Fine-tuning:** Once the RCNN model is trained, it can be fine-tuned on a specific dataset to improve its performance on that dataset.
9. **Testing:** During testing, the RCNN model is applied to new images and the object proposals are generated. The model then classifies each proposal and predicts the bounding box coordinates. The final output is a set of bounding boxes around the detected objects along with their category labels.

## 4 Experiments

### 4.1 Dataset

The dataset was downloaded from Kaggle database and consists of blood cell data. The dataset contains 12,500 augmented microscopic images of different types of blood cells in JPEG format. There are around 3000 images for each of the 4 cell types - Eosinophil, Lymphocyte, Monocyte, and Neutrophil. The cell type labels are provided as CSV files. Additionally, there are 410 original pre-augmentation images. These 410 images have 2 subtype labels (WBC vs RBC) in XML format.^1^

### 4.2 Experimental setup

(i) The proposed model's significance was demonstrated through extensive experiments in this study. These experiments utilized a personal computer with an Intel Pentium Core™ i7-6700 processor (3.7 GHz) and 16 GB of RAM. We used k-fold cross-validation to evaluate how well our approaches were being applied. The dataset was partitioned with a 60/20/20 split into training, validation, and testing sets. Ten comparable measurable sub-tests were randomly selected from the first sample. Nine sub-tests were used to train the model, and one sub-test was held to test the model after training. Ten times the process was repeated, each time using a different sub-sample for testing. The average values for the single and final results were subsequently determined by the outcomes.

(ii) The first experiment examines the efficacy of the suggested strategy by validating it across all photos.

(iii) The second experiment trains and tests the classification system for blood structure using a dataset of blood structure that was obtained. However, the suggested method was not utilized. A number of well-known feature extraction techniques were used in the experiment, including local directional pattern, local binary pattern, local directional pattern variance, wavelet transform, Hough transform, scale-invariant feature transform, curvelet transform, deformable templates, template matching, and unitary image transforms.

(iv) The concluding stage of this experiment leads to a comparison between the newly created model and the previously established novel feature extraction technique.

### 4.3 Evaluating the accuracy and processing time of the deep learning technology for image augmentation

In this section, we enhance the quality of limited data by utilizing augmentation techniques, which can effectively address the issue of overfitting. The configuration of the image augmentation technology is presented in Table 1. In the same table, where augmentation parameters are shown, these are for the training set data augmentation. Therefore, to train and test the microscopic images R-CNN model was used and then further analyzed with ResNet and GoogleNet models. We observed that R-CNN achieves better results and is less time-consuming.

**Table 1:** Setting Parameters for Feature Extraction.

Parameters	Range	Description
fill_mode	nearest	overlay the image while it is flipping
rotation_scale	1/255	Image magnification ratio
rotation_range	0.2	rotation range
horizontal_flip	1	horizontally translational range
height_shift	0.1	vertically translational range
shear_range	0.1	projected conversion range
zoom_range	0.5	the ratio of an image that zooms randomly
width_shift	0.2	horizontally translational range

### 4.4 Results and discussion

**Figure 3:**
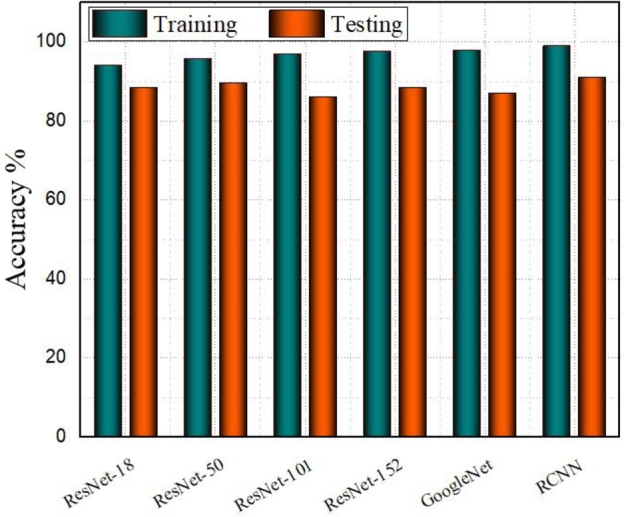
Training and Testing Accuracy of ResNet-18, ResNet-50, ResNet-101, ResNet-152, GoogleNet and RCNN Models. The Y-axis represents the accuracy percentage ranging from 0 to 100% and the X-axis lists the deep learning models. The teal-colored bars denote the training accuracy, while the orange bars represent testing accuracy.

The key results highlight the high accuracy, fast execution time, ability to handle overlaps, improved resilience with pre-processing, and overall effectiveness of the proposed approach in detecting red blood cells in microscopy images. The comparative evaluation also demonstrates its advantages over other methods. The RCNN model is a variation of the CNN model, which operates on fixed-size images. In the context of analyzing blood microscopic images, we resized the images to a uniform square size of 24x24. The training and testing results for the RCNN model indicate a high level of accuracy, with training accuracy reaching 99.06% and testing accuracy at 91.21%. The model loss values for both the training and testing phases were also low, at 2.017% and 3.312%, respectively. Additionally, the execution time was recorded as 2091 seconds and 1474 seconds for training and testing, respectively. As Fig. 3 shows the accuracy results of training and testing phases, all models have high accuracy with most of them hovering around or above 90% and RCNN has high accuracy comparatively. All the comparative models have small gaps between training and testing accuracies indicating good generalization. However, ResNet-18 and ResNet 152 have slightly larger gaps, indicating potential overfitting.

**Figure 4:**
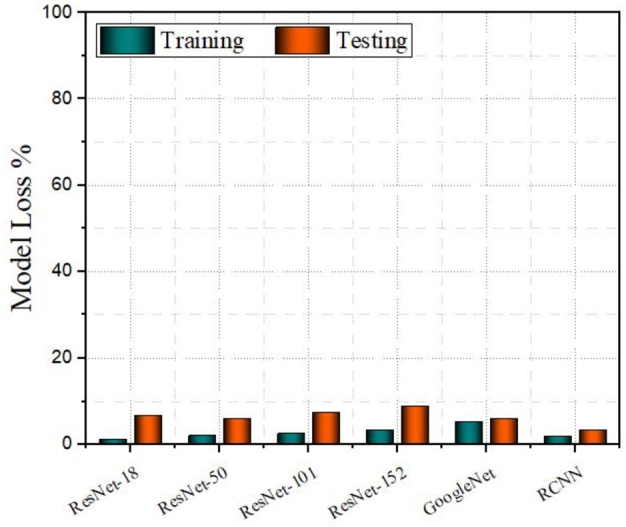
Model Loss Percentage of ResNet-18, ResNet-50, ResNet-101, ResNet-152, GoogleNet and RCNN Models. The Y-axis represents the model loss percentage ranging from 0 to 100% and the X-axis lists the deep learning models. The teal-colored bars denote the training loss, while the orange bars represent testing loss.

Fig. 4 represents the results of the model losses. Among all models, RCNN has the lowest training and testing losses. ResNet-50 and ResNet-101 also have the lowest training losses among other models after RCNN, indicating a good fit for the training data. ResNet-50 and ResNet-101. ResNet-152 has a moderate training loss, while ResNet-18 has the highest training loss among the ResNet variants but is still low in comparison to the overall scale. ResNet-18, ResNet-50, ResNet-101, and RCNN have relatively smaller gaps, suggesting better generalization.

**Figure 5:**
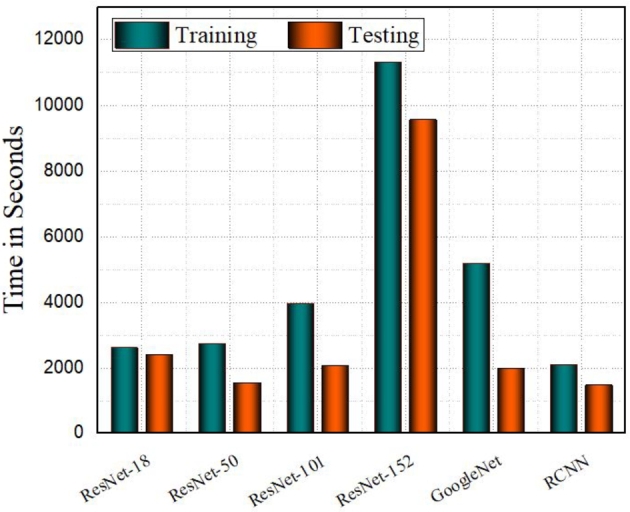
Execution time of four different ResNet models, GoogleNet, and RCNN models. The Y-axis represents the time taken in seconds ranging from 0 to 12000 and the X-axis lists the deep learning models. The teal-colored bars denote the training time, while the orange bars represent the testing time.

**Figure 6:**
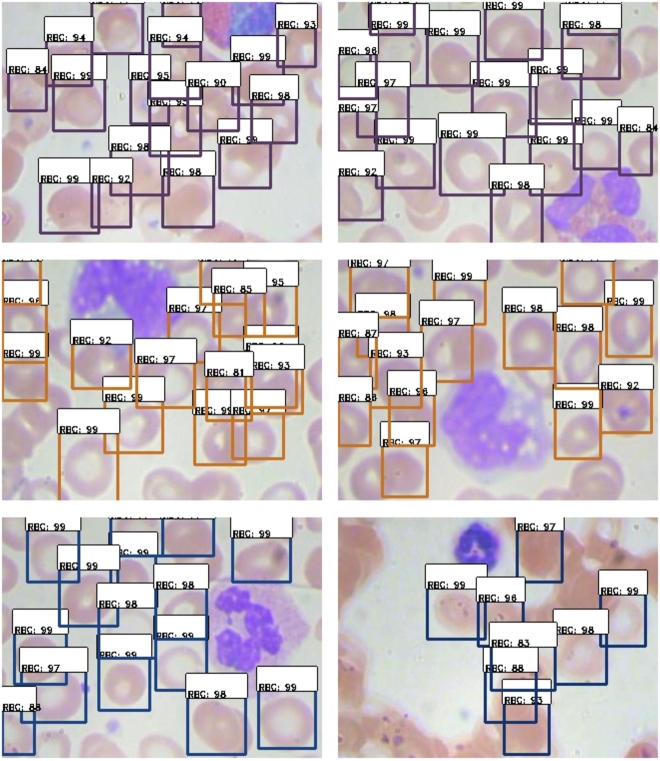
Red Blood Cells Detection. The Dark brown bounding boxes (upper two images) show correct detection, light brown bounding boxes (two images in the middle) indicate false negatives or missed cells, and dark blue boxes (two images in the bottom) show false positive detection on non-cell regions.

Fig. 5 depicts the execution time of all comparative models. ResNet-152 takes the longest time (11315 seconds) to train, and 9576 seconds to test. GoogleNet also has a high training and testing time of 5201 and 2001 respectively. In comparison to all models, RCNN took less time in execution that is 2091 for training and 1474 for testing (for a visual example of red blood detection, see Fig. 6).

**Observations:**

**ResNet Variants:** As the depth increases from ResNet-18 to ResNet-152, the training loss generally decreases, and the training accuracy generally increases. However, ResNet-152 has a slightly higher testing loss than ResNet-50 and ResNet-101. This might indicate overfitting as the model becomes deeper.

**GoogleNet:** Compared to the ResNet variants, GoogleNet has a higher training and testing loss, its accuracy, while competitive, is not the highest among the models. Its training time is notably longer than the ResNets but shorter than ResNet-152

**RCNN:** This model has the highest training accuracy of all the models listed, and its testing accuracy is competitive, However, its training and testing losses are higher compared to most ResNet variants, and its training time is between that of GoogleNet and ResNet-152. The proposed approach outpaced other standard models like ResNet-50 and GoogLeNet in accuracy by over 10-15%, proving its superiority on the same dataset.

## 5 Limitations of current research and future directions

The limitations of the current research and the next steps for creating more effective and reliable blood cell analysis technologies are highlighted by talking about these constraints and future endeavors as follows.

1. The training and evaluation datasets had a modest or narrowly varied sample size. The robustness and generalization of the model may be enhanced with a larger and more diverse dataset. Future research can apply techniques to datasets with a greater variety of cell/tissue types and imaging techniques.
2. The categories of images that could be examined were limited to specific cell types, magnification levels, or imaging modalities. The image categories could be expanded to reveal limitations. Future research can include other blood cell sub-types or blood cell classes, such as platelets.
3. The analysis was limited to cell detection and cell categorization. No more morphological characterization or analysis was carried out. Beyond only detecting and counting, future studies can characterize cells in more detail morphologically.
4. The majority of method testing took place in optimal circumstances. It's unknown how the system will perform with noisy images or other artifacts. Future studies should concentrate on evaluating the model's robustness in less perfect imaging settings and with more noise and artifacts.
5. Only comparable deep learning models were evaluated, rather than being compared to conventional image processing methods. Future research may focus on developing methods to individually reconstruct overlapping cells and improve their differentiation more effectively.
6. Overlapping cells are difficult for the model to distinguish, showing its limitations. Future studies should concentrate on making the transition from only patch analysis to comprehensive blood smear analysis.

## 6 Conclusion

For the purpose of identifying the regions of interest in microscopic pictures and detecting red blood cells based on tissue characteristics, a multi-modal strategy integrating deep learning and technology has been put forth. Three primary parts make up the proposed model: feature extraction, picture classification, and data preprocessing. Data augmentation and filtering algorithms are examples of data preparation techniques that improve dataset quality and cut down on noise. These techniques aid in boosting the model's accuracy and computing efficiency. Feature extraction by integrating biosensors and deep learning model is the process of extracting valuable features from a dataset that can be used to classify images. In this method, feature extraction is done using the deep learning method RCNN, and performance against ResNet and GoogleNet is evaluated. In order to determine the regions of interest based on tissue attributes, classification is carried out. The proposed model was highly accurate and can be used to diagnose and treat a variety of illnesses. Overall, the approach is effective in achieving high accuracy while consuming less time. This multi-modal approach is a promising direction for improving the diagnosis and treatment of various medical conditions in microscopic images.

## CRediT authorship contribution statement

**Riaz Ullah Khan:** Visualization, Methodology, Funding acquisition, Formal analysis, Data curation. **Sultan Almakdi:** Resources, Funding acquisition, Formal analysis. **Mohammed Alshehri:** Writing – review & editing, Project administration, Investigation, Data curation. **Amin Ul Haq:** Validation, Software, Conceptualization. **Aman Ullah:** Methodology, Data curation, Conceptualization. **Rajesh Kumar:** Writing – review & editing, Visualization, Validation, Software, Formal analysis, Data curation.

## Declaration of Competing Interest

The authors declare the following financial interests/personal relationships which may be considered as potential competing interests:

Riaz Ullah Khan reports article publishing charges and statistical analysis were provided by Yangtze Delta Region Institute (Huzhou), University of Electronic Science and Technology of China. Sultan Almakdi reports article publishing charges was provided by Najran University. If there are other authors, they declare that they have no known competing financial interests or personal relationships that could have appeared to influence the work reported in this paper.

## Footnotes

1 https://www.kaggle.com/paultimothymooney/blood-cells/home.
